# A GORTEC survey on low-risk CTV-P2 delineation in head and neck cancers^☆^

**DOI:** 10.1016/j.ctro.2025.100980

**Published:** 2025-05-20

**Authors:** Michel Lapeyre, Yoann Pointreau, Marc Alfonsi, Pierre Boisselier, Julian Biau, Pierre Blanchard, Joël Castelli, Pierre Graff, Florence Huguet, Laurent Martin, Séverine Racadot, Xu Shan Sun, Yungan Tao, Jean Bourhis, Juliette Thariat

**Affiliations:** a Department of Radiation Therapy, Jean Perrin Centre, 58 Rue Montalembert, BP 5026, 63011 Clermont Ferrand Cedex 1, France; b Department of Radiation Therapy, Inter-Regional Institute of Oncology (ILC, Institut inter-régionaL de Cancérologie), Jean Bernard Centre, Sarthe Oncology Centre 64 Rue de Degré 72000 Le Mans, France; c Department of Radiation Therapy, Sainte-Catherine Institute – Avignon-Provence Cancer Institute, CS 80005, 250 Chemin de Baigne Pieds, 84918 Avignon Cedex 9, France; d Department of Radiation Therapy, Montpellier Regional Cancer Institute, Parc Euromédecine, 208 rue des Apothicaires, 34090 Montpellier, France; e Department of Radiation Therapy, Gustave Roussy Institute, 114 Rue Edouard Vaillant, 94805 Villejuif, France; f Department of Radiation Therapy, Eugène Marquis Centre, Av. de la Bataille Flandres-Dunkerque CS 44229, 35000 Rennes, France; g Department of Radiation Therapy, Curie Institute, 26 rue d’Ulm, 75248 Paris Cedex 05, France; h Department of Radiation Therapy, Tenon Hospital, 4 rue de la Chine, 75020 Paris, France; i Guillaume Le Conquérant Centre, 61 Rue Denfert Rochereau, 76600 Le Havre, France; j Department of Radiation Therapy, Léon Berard Centre, 28 Rue Laennec, 69008 Lyon, France; k Department of Radiation Therapy, Franche-Comté North Hospital in Montbéliard & Besançon CHRU (Centre hospitalier régional universitaire [Regional University Hospital]), 1 Rue Henri Becquerel, 25200 Montbéliard, France; l Department of Radiation Therapy, Lausanne University Hospital (CHUV, Centre Hospitalier Universitaire Vaudois), Rue du Bugnon 46, 1005 Lausanne, Switzerland; m Department of Radiation Therapy, Comprehensive Cancer Centre François Baclesse, 3 Avenue du General Harris, 14000 Caen, France

**Keywords:** Cancer, Head and neck, Delineation, CTV definition, Consensus Guidelines

## Abstract

**Purpose:**

An international consensus was established in 2018 to standardise practice using geometric (5 + 5 mm) expansion around GTV-P for definitive radiotherapy of squamous cell carcinomas of the head and neck (HNC). The GORTEC (French HNC Oncology and Radiotherapy Group) conducted a survey to assess the level of agreement about CTV-P2 delineation using a “formalised consensus method”.

**Methods:**

The 32 proposals of the 2018 consensus on CTV-P2 and 6 additional GORTEC proposals were submitted to 13 GORTEC radiation oncologists (RO). Proposals were rated as “suitable” for median scores ≥7, “unsuitable” for scores ≤3.5 or “uncertain.” The degree of agreement was high (≥85 %), moderate (75–84 %) or low (<75 %). Suitable proposals were reviewed by 40 other RO for final recommendations.

**Results:**

The 2018 proposals were “uncertain” with low degrees of agreement (41.5–69 %), except for T1 tumors, which had 89 % agreement. Five out of 6 GORTEC proposals were “suitable” and one “uncertain.” The final recommendation was “suitable and to be retained” by 97.5 % of RO, as follows: To obtain CTV-P2, GORTEC recommends applying a “geo-anatomical” approach. Using the geometric concept, 10 mm-isotropic margins are applied to the GTV, for all locations but the hypopharynx (10 mm antero-posterior, laterally and 15 mm craniocaudally). CTV-P2 is further modified using the anatomical concept (anatomical barriers, dissemination routes) and accounting the benefit/risk balance and proximity of organs at risk.

**Conclusion:**

The GORTEC survey derived from the 2018 international CTV-Ps delineation consensus suggests a “geo-anatomical” approach for the delineation of CTV-P2 in HNC.

## Highlights

• Geometric margins around the GTV-P help to standardise CTV-P2 delineation for definitive radiotherapy of HNC
• A geo-anatomical definition of CTV P2 may be needed to account for dissemination routes and anatomical barriers.
• Using a “formalised consensus method”
• 97.5 % of GORTEC radiation oncologists on a geo-anatomical CTV-P2 delineation, also accounting for the proximity of organs at risk.

## 1 Introduction

Delineation of the primary tumor target volumes (CTV-P) of squamous cell carcinomas of the larynx, hypopharynx, oropharynx, oral cavity (HNC) varies significantly [1]. An international delineation consensus [2] suggested that the CTV-Ps be delineated according to the geometrical concept of the Danish HNC Group (DAHANCA) in the definitive radiotherapy setting [3]. Low-dose volumes (CTV-P2) were standardised using isotropic 5 + 5 mm expansions around macroscopic tumor volumes (GTV-P), except for 15 mm craniocaudal expansions for the hypopharynx. These margins were adapted according to tumor location and stage (T classification). The high-dose volume (CTV-P1) was defined using 5 mm margin expansions around GTV-P then resizing CTV-P1 within CTV-P2. In some situations with small T1 tumors, the consensus proposed to delineate only CTV-P1, without a CTV-P2. Finally, each situation had to be adapted on a case-by-case basis. Other authors advocated that the use of the geometrical method was recent and insufficiently evaluated to fully validate this approach from a clinical standpoint [4,5] and the consensus was based on experts’ opinions rather than a formalised consensus methodology [5]. The Head and Neck Oncology and Radiotherapy Group (GORTEC) was using a more “anatomical method”, based on knowledge of tumor extension pathways and anatomical barriers [[6], [7], [8]], which allowing the delineation of CTV P2, as outlined in Appendix 1 and 2.

The aim of the current GORTEC survey was to assess the recommendations of the 2018 international consensus for the delineation of low-risk CTV of the primary tumor (CTV-P2) within its members. It was proposed to enrich these recommendations with additional recommendations based on literature and expertise, and using a “formalised consensus” methodology [9].

## 2 Methods and materials

The study evaluated the proposals of the international consensus based on the international experts’ opinions and additional proposals from GORTEC experts and data from the literature. A “formalised consensus” methodology, developed from the Delphi, RAND/UCLA methods and from consensus conferences was used [[10], [11], [12], [13]]. Information present in the 2018 consensus was summarised. The methodology aimed to formalise the degree of agreement between experts where the opinion would not be unanimous as could be the case when the data from literature have low or contradictory levels of evidence [9]. The results were also reported using the “percentage of agreement” between experts [14].

The formal consensus process included 5 steps: 1) summary of the literature, 2) analysis of the international consensus proposals 3) drafting of proposals during a GORTEC meeting to be submitted to a group of experts (2 scoring phases), 4) drafting of an initial version of recommendations from the 2 scoring phases, 5) scoring of the recommendations proposed to a reading group and writing of the recommendations. Three groups were defined (a steering committee with a project manager, a first group of experts for phase 1 of the scoring (group 1) then a second group for phase 2 of the scoring (group 2) and a reading group (group 3).

The steering group was comprised of three members. After drafting the summary of the bibliographical data, it established a questionnaire containing proposals on the delineation of CTV-P2 in order to submit it to group 1. Scores and degree of agreement were analysed. Proposals with “strong agreement” were deemed to be acceptable and were validated automatically. Proposals deemed as ambiguous as to their formulation, could be modified and proposed for a second phase of scoring to a more limited group 2. This was necessary when the results showed “indecisive/uncertainty” or if there was “no consensus obtaining at least a relative agreement.” The analysis of the results of the second round of scoring allowed for a degree of tolerance with possibly the removal of an extreme proposal, in order to avoid systematically rejecting certain proposals and blocking the process in accordance with the “formalised consensus” methodology.

Scoring group 1 was comprised of 13 members. These were senior member Radiation Oncologists, experts and dedicated in radiotherapy of HNC. These members were asked to examine the proposals of the international consensus and to read the review of the literature that was provided before scoring. Scoring group 2 was comprised of 9 members with the same pre-requisite. All of the proposals and answers were collected via an online secured encrypted GDPR (General Data Protection Regulation)-compliant questionnaire (https://www.easy-crf.com/CTV_GORTEC).

The proposals were scored using a numeric gradual scale from 1 to 9; from 1 for “entirely unsuitable”, to 9 “entirely suitable” proposals, a score of 5 corresponding to “indecisive”. The formal analysis relied on the median and extreme scores to define a “suitable or not” nature of the proposal (Table 1). The results were also reported as in [14], as “in agreement” defined by a score >5, with high ≥85 %; moderate: 75–84 % or low <75 % in agreement.

**Table 1:** Definitions* of agreement levels based on median value and distribution of quotes.

Estimation of the proposal	Type of agreement obtained	Median of the scoring	Extremes of the scoring
Suitable	Strong Agreement	≥7	(7–9)
	Relative agreement	≥7	(5–9)
Unsuitable	Strong Agreement	≤3	(1–3)
	Relative agreement	≤3.5	(1–5)
Uncertain	Indecisive	4 ≤ median ≤ 6.5	(1–9)
	Absence of consensus	Other situations	

*(quotes were used to define “suitable”, “uncertain” or “unsuitable” proposals according to the “formalised consensus” method).

The first scoring phase included a 2-part questionnaire: Part 1 consisted of the proposals for the delineation of CTV-P2 of the international consensus, pages from 10 to 20 [2], taken point by point and without modification (32 proposals). These 32 proposals were further grouped by T stage. However, T1 glottic tumors with a proposal for delineation of CTV-P1 without CTV-P2 were individualised as well as T1 superficial tumors excluding glottic and T2 superficial glottic and supraglottic tumors for which a delineation of CTV-P1 without CTV-P2 was proposed [2]. Part 2 contained the 6 additional GORTEC proposals.

Based on an analysis of the results of the first round of scoring, the proposals could be modified by the steering group for ambiguous phrasing and then subjected to a second scoring phase. For the international consensus proposals, no reformulation of validated non-modifiable proposals was allowed.

Using the results of the 2 scoring phases, the steering group proposed an initial version of 2 recommendations. The wording and formulation of the recommendations complied with the rules of the “formalised consensus” method. (Table 2).

**Table 2:** Formulation rules for the initial recommendation; at the end of the 2 scoring steps for the reading group according to the “formalised consensus” method.

Estimation of the proposal		Type of possible formulation
Suitable		It is recommended to…
Uncertain	Indecisive	The basis of current knowledge does not make it possible to make a conclusion concerning
	Absence of consensus	Deletion of the proposal or The basis of current knowledge and in the absence of a consensus, it cannot be recommended…
Unsuitable		It is recommended to not… or Deletion of the proposal

The first version of the recommendations was submitted by means of the same online system for opinion of the reading group comprised of 40 additional GORTEC members, also specialized in head and neck radiotherapy. It was accompanied by the scientific argumentation and summary tables of the results of the 2 rounds of scoring. The members of the reading group established their scoring according to the same rules as in earlier rounds.

The responses of the reading group were used to rank the proposals and to formulate a final version. According to the “formalised consensus” method, the recommendation was defined as “suitable and retained” if there were at least 90 % of the responses from the reading group in the 5 to 9 interval. If the reading group did not agree with the initial proposal (<90 % of the responses of the reading group in the 5 to 9 interval), it was possible to propose either a “possible modification” of the proposal according to the comments or “rejection of the recommendation”.

## 3 Results

*Step 1: first scoring phase (*Table 3, Table 4*)*.

**Table 3:** Results of scoring step 1 (13 experts) for the 32 proposals for delineating ctv-p of the international consensus (geometrical method, CTV-P1 = GTV-P + 5 mm; CTV P2 = GTV-P + 10 mm; 15 mm craniocaudal for HP; “Guidelines for the delineation of the primary tumour CTV, pp10-20” [2].

Proposals grouped by T stage and T location (OC, OP, HP, L)* Number of proposals listed (page numbers of the international consensus) [2]	Methods						
	“Percentages of agreement” method				“Formalised consensus” method		
	Agreement	Indecisive	Disagreement	Degree of agreement**	Median (extreme)	Proposals	Degree of agreement
T1 glottic (CTV-P1 alone) 1 proposal (pp. 10)	62 %	15 %	23 %	low	8 (1–9)	Uncertain	Absence of consensus
T1 glottic (CTV-P1 + CTV-P2) 1 proposal (pp. 10)	77 %	8 %	15 %	moderate	9 (2-9)	Uncertain	Absence of consensus
Superficial T1 excluding G (CTV-P1 alone) 5 proposals (pp. 12–14, 16, 19)	41.5 %	14 %	44.5 %	low	5 (1–9)	Uncertain	Absence of consensus
T1 excluding G (CTV-P1 + CTV-P2) 5 proposals (pp. 12–14, 16, 18)	89 %	8 %	3 %	high	7 (3–9)	Uncertain	Absence of consensus
Superficial T2 G / supraG (CTV-P1 alone) 2 proposals (pp. 10, 12)	69 %	4 %	27 %	low	7 (1–9)	Uncertain	Absence of consensus
T2 (CTV-P1 + CTV-P2) 6 proposals (pp. 10, 12–14, 16, 19)	67 %	18 %	15 %	low	7 (1–9)	Uncertain	Absence of consensus
T3 (CTV-P1 + CTV-P2) 6 proposals (pp. 10, 12, 14, 15, 17, 19)	64 %	9 %	27 %	low	7 (1–9)	Uncertain	Absence of consensus
T4 (CTV-P1 + CTV-P2) 6 proposals (pp. 11, 13–15, 17, 19, 20)	63 %	8 %	29 %	low	7 (1–9)	Uncertain	Absence of consensus

Legend:

*L: larynx; SupraG: supraglottic; G: glottic larynx; HP: hypopharynx, OP: oropharynx; OC: oral cavity.

**high agreement ≥85 agreement; moderate (75–84 % agreement); low <75 % agreement.

**Table 4:** Results of scoring step 1 for the 6 GORTEC proposals on CTV-P2 delineation (13 experts).

GORTEC proposals	Methods						
	“Percentages of agreement” method				“Formalised consensus” method		
	Agreement	Indecisive	Disagreement	Degree of agreement*	Median (extreme)	Proposal	Degree of agreement**
The consensus is a step forward in unifying practices	84.6 %	7.7 %	7.7 %	Moderate	7 (4–9)	Uncertain	Absence of consensus
Data are insufficient to validate the systematic application of the geometrical approach (5 + 5 mm or 5 + 10 mm for the hypopharynx)	92 %	0 %	8 %	High	9 (3–9)	Uncertain	Absence of consensus
Some recurrences occur beyond 10 mm from the edge of the GTV and should be taken into account	84.6 %	7.7 %	7.7 %	Moderate	9 (4–9)	Uncertain	Absence of consensus
The post-resection margin shrinkage can be used to propose extending the delineation of CTV-P2 beyond 10 mm of the GTV (tongue, inner cheek, pharynx for example)	100 %	0 %	0 %	High	8 (6–9)	Suitable	Relative agreement
To obtain CTV-P2, application of the geo-anatomical solution should be preferred. It should start with the geometrical concept (GTV + 10 mm; 15 mm craniocaudally for the hypopharynx) then extend the delineation with the anatomical concept of the tumour dissemination routes while also taking account of the risk of complications linked to the dose to OAR (constrictor muscles of the pharynx, jaw, etc.)	100 %	0 %	0 %	High	9 (6–9)	Suitable	Relative agreement
It is possible to treat only CTV-P1 for small superficial T1 tumours (excluding the glottic larynx)	46 %	8 %	46 %	Low	5 (1–9)	Uncertain	Indecisive

Legend: *high agreement ≥ 85 agreement; moderate (75–84 % agreement); low <75 % agreement.

**proposals with a relative agreement, absence of consensus or indecisive must go through a second round.

Most of the proposals were classified as “uncertain” and could not reach a consensus. The medians varied from 5 to 7 with extremes from 1 to 9. The rate of agreement varied from 62 to 69 % (low degree of agreement). The T1 superficial tumors excluding the glottis for which a delineation of CTV-P1 without CTV-P2 was proposed obtained a rate of agreement of only 41.5 %. Alternatively, delineating the non-glottic T1 tumors with both CTV-P1 and CTV-P2 obtained a rate of agreement of 89 % (high degree of agreement) (Table 3).

Of the 6 additional GORTEC CTV-P2 proposals, four were classified as “uncertain” and two as “suitable” with relative agreement. The rate of agreement for the “percentages of agreement” method varied from 46 to 100 % with a low to high degree of agreement (Table 4).

*Step 2: 2nd scoring phase (*Table 5*)*.

**Table 5:** Results of scoring step 2 for the 6 GORTEC proposals. the GORTEC recommendations were reformulated after analysis of the results of phase 1 (9 experts).

GORTEC proposals	Methods						
	“Percentages of agreement” method				“Formalised consensus” method		
	Agreement	Indecisive	Disagreement	Degree of agreement*	Median (extreme)	Proposal	Degree of agreement**
Obtaining a consensus is a step forward in unifying practices	100 %	0 %	0 %	High	9 (7–9)	Suitable	Strong Agreement
Data are still insufficient to validate the systematic application of the geometrical approach (5 + 5 mm or 5 + 10 mm)	63 %	37 %	0 %	Low	8 (5–9)	Suitable	Relative agreement
There is a number of recurrences beyond 10 mm from the edge of the GTV that have to be taken into account	100 %	0 %	0 %	High	8 (7–9)	Suitable	Strong Agreement
In light of the post-resection margin shrinkage (tongue, internal face of the cheek, parapharynx for example), the margin of extension beyond the GTV of 10 mm in order to obtain the CTV-P2 can be insufficient	87 %	13 %	0 %	Moderate	8 (5–9)	Suitable	Relative agreement
To obtain CTV-P2, application of the “geo-anatomical” approach is preferred, namely starting with the geometrical concept (GTV + 10 mm; 15 mm craniocaudally for hypopharynx) then extending the delineation with the anatomical concept (anatomical barriers, tumour dissemination routes) by taking account of the risk benefit ratio (tumour control/toxicity).	100 %	0 %	0 %	High	9 (7–9)	Suitable	Strong Agreement
It is possible to treat only CTV-P1 for small superficial T1 tumours (excluding the glottic larynx)	33 %	11 %	56 %	Low	3 (1–9)	Uncertain	Absence of consensus

Legend: *high agreement ≥85 agreement; moderate (75–84 % agreement); low <75 % agreement.

**A relative or strong degree of agreement can be subject to validation or recommendation

After debate during bi-annual GORTEC meeting, a second round of scoring was proposed for the GORTEC proposals deemed as ambiguous in their formulation. These modified proposals were subjected to a second online questionnaire (9 experts). Five proposals were deemed to be “suitable” with medians varying from 8 to 9 (extremes from 5 to 9). The degrees of agreement varied from “low to strong” according to the proposal with a rate of agreement that varied from 63 to 100 %. The following CTV-P2 proposal obtained a rate of agreement of 100 % (“suitable” proposal with strong agreement): “To obtain CTV-P2, finally GORTEC proposes applying a ”geo-anatomical“ approach. Using the geometric concept, 10 mm isotropic margin expansions are applied to the GTV for all locations, but the hypopharynx where 10 mm antero-posterior and lateral and 15 mm craniocaudal margin expansions are applied. CTV-P2 is further modified using the anatomical concept (anatomical barriers, dissemination routes) by taking account of the benefit/risk balance according to the proximity of organs at risk*”*. In contrast, the 2018 recommendation stating that it was possible to treat only CTV-P1 for small superficial tumors excluding the glottic larynx was deemed “uncertain” with a median at 3 [[1], [2], [3], [4], [5], [6], [7], [8], [9]] and a rate of agreement at 33 % (low agreement).

*Step 3: reading phases of the initial proposals (*Table 6*)*.

**Table 6:** Results of the reading phase of the gortec proposals (40 readers).

GORTEC proposals	Methods					
	“Percentages of agreement” method				“Formalised consensus” method	
	Agreement	Indecisive	Disagreement	Degree of agreement*	Rate of response between 5 and 9	Recommendation **
On the basis of current knowledge and in the absence of a consensus, it cannot be recommended to treat only CTV P1 for small superficial tumours excluding glottic	67.5 %	7.5 %	25 %	Low	75 %	To be rejected
To obtain CTV-P2, application of the “geo-anatomical” approach is preferred, namely starting with the geometrical concept (GTV + 10 mm; 15 mm craniocaudally for hypopharynx) then extending the delineation with the anatomical concept (anatomical barriers, tumour dissemination routes) by taking account of the risk benefit ratio (tumour control/toxicity).	95 %	2.5 %	2.5 %	High	97.5 %	Suitable and to be retained

Legend: *high agreement ≥85 agreement; moderate (75–84 % agreement); low <75 % agreement.

**Suitable recommendation and to be retained if the responses obtained in the interval [[5], [6], [7], [8], [9]] are ≥90 %.

The initial CTV-P2 recommendations were presented to the GORTEC group and answered electronically by 40 readers and presented at the June 2022 GORTEC meeting. The first proposal stated that based on current knowledge and lack of consensus, it could not be recommended to treat only CTV-P1 for small superficial tumors (excluding the glottic larynx*).* It was “rejected” due to a response rate between 5 and 9 of 75 %. Some reviewers recommended that delineation should comprised both a CTV-P1 and a CTV-P2, and others that radiation oncologist be left to decide on a case-by-case basis. The second proposal for the delineation of CTV-P2 was as follows: GORTEC proposes applying a “geo-anatomical” approach to obtain CTV-P2. Using the geometric concept, 10 mm isotropic margin expansions are applied to the GTV for all locations but the hypopharynx where 10 mm antero-posterior and lateral and 15 mm craniocaudal margin expansions are applied. CTV-P2 is further modified using the anatomical concept (anatomical barriers, dissemination routes) by taking account of the benefit/risk balance according to the proximity of organs at risk*.* This proposal obtained a 97.5 % rate for responses between 5 and 9 and was considered as “suitable and to be retained”. Table 7 summarizing all the step 1 score agreements of the 32 proposals for the International Consensus CTV-P delineation is included.

**Table 7:** Table summarizing all the step 1 score agreements of the 32 proposals for delineating CTV-P of the international consensus*.

Proposals grouped by T location. Proposals listed (page numbers of the international consensus) (2)		Methods						
		“Percentages of agreement” method				“Formalised consensus” method		
		Agreement	Indecisive	Disagreement	Degree of agreement**	Median (extreme)	Proposals	Degree of agreement
Glottic pp 10	T1 glottic (CTV-P1 alone)	62 %	15 %	23 %	low	8 (1–9)	Uncertain	Absence of consensus
	T1 glottic (CTV-P1 + CTV-P2)	77 %	8 %	15 %	moderate	9 (2–9)	Uncertain	Absence of consensus
	T2 glottic (CTV-P1 alone)	54 %	0 %	46 %	low	6 (1–9)	Uncertain	Indecisive
	T2 glottic (CTV-P1 + CTV-P2)	70 %	15 %	15 %	low	7 (2–9)	Uncertain	Absence of consensus
	T3 glottic (CTV-P1 + CTV-P2)	77 %	0 %	23 %	moderate	7 (1–9)	Uncertain	Indecisive
	T4 glottic (CTV-P1 + CTV-P2)	69 %	8 %	23 %	low	8 (1–9)	Uncertain	Absence of consensus
Supraglottic pp 12	T1 Supraglottic (CTV-P1 alone)	54 %	0 %	46 %	low	6 (1–9)	Uncertain	Indecisive
	T1 Supraglottic (CTV-P1 + CTV-P2)	92 %	0 %	8 %	high	7 (3–9)	Uncertain	Absence of consensus
	T2 Supraglottic (CTV-P1 alone)	84 %	8 %	8 %	moderate	8 (3–9)	Uncertain	Absence of consensus
	T2 Supraglottic (CTV-P1 + CTV-P2)	77 %	15 %	8 %	moderate	7 (3–9)	Uncertain	Absence of consensus
	T3 Supraglottic (CTV-P1 + CTV-P2)	69 %	8 %	23 %	low	8 (1–9)	Uncertain	Absence of consensus
	T4 Supraglottic (CTV-P1 + CTV-P2)	69 %	0 %	31 %	low	8 (1–9)	Uncertain	Absence of consensus
Sub-glottic pp 13	T1 Subglottic (CTV-P1 alone)	46 %	8 %	46 %	low	5 (1–9)	Uncertain	Indecisive
	T1 Subglottic (CTV-P1 + CTV-P2)	92 %	0 %	8 %	High	7 (3–9)	Uncertain	Absence of consensus
	T2 Subglottic (CTV-P1 + CTV-P2)	77 %	15 %	8 %	moderate	7 (3–9)	Uncertain	Absence of consensus
	T3 Subglottic (CTV-P1 + CTV-P2)	62 %	15 %	23 %	low	7 (1–9)	Uncertain	Absence of consensus
	T4 Subglottic (CTV-P1 + CTV-P2)	62 %	15 %	23 %	low	7 (1–9)	Uncertain	Absence of consensus
Hypopharynx pp 14	T1 Hypopharynx (CTV-P1 alone)	31 %	15 %	54 %	low	3 (1–9)	Uncertain	Absence of consensus
	T1 Hypopharynx (CTV-P1 + CTV-P2)	84 %	8 %	8 %	moderate	8 (3–9)	Uncertain	Absence of consensus
	T2 Hypopharynx (CTV-P1 + CTV-P2)	69 %	8 %	23 %	low	7 (1–9)	Uncertain	Absence of consensus
	T3 Hypopharynx (CTV-P1 + CTV-P2)	69 %	8 %	23 %	low	7 (1–9)	Uncertain	Absence of consensus
	T4 Hypopharynx (CTV-P1 + CTV-P2)	69 %	8 %	23 %	low	7 (1–9)	Uncertain	Absence of consensus
Oropharynx pp 16	T1 Oropharynx (CTV-P1 alone)	38 %	23 %	29 %	low	5 (1–9)	Uncertain	Indecisive
	T1 Oropharynx (CTV-P1 + CTV-P2)	92 %	0 %	8 %	high	8 (3–9)	Uncertain	Absence of consensus
	T2 Oropharynx (CTV-P1 + CTV-P2)	54 %	15 %	31 %	low	6 (1–9)	Uncertain	Indecisive
	T3 Oropharynx (CTV-P1 + CTV-P2)	62 %	8 %	30 %	low	6 (1–9)	Uncertain	Indecisive
	T4 Oropharynx (CTV-P1 + CTV-P2)	54 %	15 %	31 %	low	8 (1–9)	Uncertain	Absence of consensus
Oral Cavity pp 18	T1 Oral Cavity (CTV-P1 alone)	38 %	24 %	38 %	low	5 (1–9)	Uncertain	Indecisive
	T1 Oral Cavity (CTV-P1 + CTV-P2)	84 %	8 %	8 %	moderate	8 (3–9)	Uncertain	Absence of consensus
	T2 Oral Cavity (CTV-P1 + CTV-P2)	54 %	31 %	15 %	low	7 (2–9)	Uncertain	Absence of consensus
	T3 Oral Cavity (CTV-P1 + CTV-P2)	46 %	16 %	38 %	low	5 (1–9)	Uncertain	Indecisive
	T4 Oral Cavity (CTV-P1 + CTV-P2)	54 %	0 %	46 %	low	7 (1–9)	Uncertain	Absence of consensus

Legend: *(geometrical method, CTV P1 = GTV-P + 5 mm; CTV P2 = GTV-P + 10 mm; 15 mm craniocaudal for HP; “Guidelines for the delineation of the primary tumour CTV, pp10-20″(1)); **high agreement ≥ 85 agreement; moderate (75–84 % agreement); low < 75 % agreement.

^a^Grégoire V, Evans M, Le QT, Bourhis J, Budach V, Chen A, et al. Delineation of the primary tumour Clinical Target Volumes (CTV-P) in laryngeal, hypopharyngeal, oropharyngeal and oral cavity squamous cell carcinoma: AIRO, CACA, DAHANCA, EORTC, GEORCC, GORTEC, HKNPCSG, HNCIG, IAG-KHT, LPRHHT, NCIC CTG, NCRI, NRG Oncology, PHNS, SBRT, SOMERA, SRO, SSHNO, TROG consensus guidelines. Radiother Oncol J Eur Soc Ther Radiol Oncol. janv 2018;126(1):3–24.

## 4 Discussion

The international consensus, through its geometrical approach, is a step forward in unifying practices in the delineation of CTV-P2 for HNC [2,15]. The GORTEC survey intended to assess the degree of agreement to this consensus in routine practice, so as to adopt it as it or customize it to particular situations if needed. Historically, the determination of CTV-P2 was established using an anatomical approach using tumor dissemination pathways and anatomical barriers [[6], [7], [8]]. The geometrical method was more recent and was initially defined by the DAHANCA. It allows volumes to be standardised [3,15]. The GORTEC delineation proposal for low-risk tumor target volumes (CTV-P2) used the geometrical approach supplemented by an anatomical approach called “geo-anatomical approach”. This was assessed on a formalized consensus approach among experts, and was submitted to external readers.

Two studies evaluated the difference in volume of CTV-P between the geometrical and anatomical methods. Gregoire et al. [16] proposed a delineation comparison for a T3N2b base of tongue carcinoma and a T2N0 right vocal cord carcinoma. Three methods were compared: anatomical or “French guideline”, DAHANCA and international consensus. For the base of tongue tumor, the CTV-P2s were respectively 140.6, 75.6 and 74.1 ml and for the CTV-P1, 54.6, 41.3 and 40.9 ml. For the larynx, the CTV-P2s were respectively 54, 13 and 11.4 and the CTV-P1s, 5, 4.3 and 3.9 ml. Bondue et al. [17] proposed four laryngeal cancer cases T2-3NO with the 2 methods (anatomical or “French guideline” and international consensus). The average ratios of the CTV-Ps were for CTV-P2 3.23 (SD: 1.06) and for CTV-P1 1.07 (SD: 0.13). The CTV-P1 volumes were identical but the anatomical method resulted in larger low risk CTV-P2 volumes by a factor of 2 to 3. The geo-anatomical approach increases the low-risk volume due to inclusion of the tumor dissemination pathways. It can be adjusted to account for the benefit/risk balance according to the proximity of the organs at risk.

The geometrical method defines a 10 mm (5 + 5) distance beyond the GTV-P to create the CTV-P2 based on studies assessing microscopic extensions on excised tissues [2]. In studies of 10 to 57 patients [[18], [19], [20], [21], [22]], primarily including oral cavity, oropharynx and hypopharynx tumors, the maximum microscopic extensions were of 18 mm for cancers of the tongue and 20 mm for cancers of the hypopharynx. In 95 % of cases, tumoral extensions were at a distance of ≤ 15 mm from the hypopharyngeal GTV-P and 4–12 mm for the tongue. However, in these studies [[18], [19], [20], [21], [22]], post-resection margin shrinkage (estimated between 10 to 25 % [[23], [24], [25], [26], [27]] was not reported and patient numbers were low. Shrinkage was more substantial for smaller tumors and varied with tumor location (more substantial for the internal face of the cheek and tongue). In order to delineate CTV-P2, a 10 mm distance around the GTV seemed to be the minimum distance and for a certain number of patients, this distance was underestimated according to the tumor location, the tumor size and tissue retraction. However, the relevance of delineating CTV-P2s (low risk) in addition to CTV-P1 (high risk) has been questioned as dose gradient around CTV-P1 results in a “CTV-P2” dose. Corkum et al. [28] showed that adding a CTV-P2 structure around the CTV-P1 using the 5 + 5 mm rule had little dosimetric impact compared to planning on CTV-P1 only. However, this may vary substantially with constraints to surrounding organs at risk. Delineating the CTV-P2 controls the dose gradient and may errors and failures [29].

The clinical results of the geometrical method are still insufficiently reported. Zukaukaite *et al.* [30] evaluated the patterns of relapse of 119 patients out of 1,576 patients treated with curative intent with the geometrical concept in two out of three Danish centres. After coregistration of the recurrence on the dosimetric scanner, a “point of origin” was created, located at the barycentre of the recurrence CT and planning CT. The distance between the “point of origin” of the recurrence and the edge of the GTV-P of the initial dosimetric scanner was measured: 49 % of the recurrences were outside the GTV-P, 17 % outside the isodose 95 % and 24 % were at more than 1 cm from the edge of the GTV-P. Although the number of recurrences was low beyond 1 cm in this study, the geo-anatomical approach might better cover these zones of potential recurrence.

As for the suppression of the delineation of CTV-P2 in T1 tumors and delineation of CTV-P1 only, the risk of local recurrence remains to be evaluated in particular for the pharynx. As is, the 2018 proposal may seem controversial, except for purely glottic tumors [4]. The GORTEC survey found a rate of agreement of 41.5 % for this proposal. The GORTEC proposal therefore stated that it cannot be “recommended, except for glottic HNC, to treat only CTV-P1 even for superficial tumors”. This GORTEC statement obtained a rate of agreement from the reading group of 67.5 % with scores between 5 and 9 in 75 % of the respondents. The proposal did not obtain the necessary score of over 90 % to be proposed as a recommendation according to the “formalised consensus” methodology, but emphasizes that there remains some controversy.

Several interobserver studies have been conducted to assess tumor delineation variability and measure the impact of guidelines on delineation variability. Van Der Veen *et al.* [31] studied variability in CTV-P and N delineation for 5 HNC cases at 14 centers in 2017. Three out the 14 centres proposed both delineation of a high dose CTV-P1 and a low dose CTV-P2. The analysis conducted on CTV-P1 in all centres showed heterogeneities in practices (median DICE Similarity Coefficient (DSC) of 0.67 to 0.82) despite the use of a previous international consensus on nodal volumes. In Bollen’s study, inter-observer variability of CTV-P delineation for 5 locations at 17 centres was reduced with use of the international consensus for the CTV-P2 [15]: the median DSC improved from 0.73 in 2017 to 0.83 in 2022. There was indeed no difference (DSC > 0.9) for CTV-P1, as the latter was directly linked to the delineation of the GTV-P, which was delineated beforehand in the study. Noticeably, for CTV-P2, such as in laryngeal tumors, 60 % of the observers reported a need for additional guidelines. In Cardenas’ study of 4 oropharyngeal HNC submitted to 24 radiation oncologists for the delineation of GTV-P using MRI, the DSC was 0.77 [32] suggesting that quality control is necessary also for the GTV-P. Although guidelines contribute to harmonisation of practice, for CTV-P2 [15] and GTV-P [32], difficulties with delineation remain. In addition, the risk of applying the geometrical (5 + 5 mm) approach might result in an increased tumor failure risk. Correlative analyses of irradiation volumes, failures and corresponding anatomy and dissemination pathways remain indispensable to validate new strategies [[6], [7], [8],^33^,^34^]. A large study was recently conducted by the DAHANCA group in almost 2000 patients with pharyngeal, and laryngeal squamous cell carcinomas treated with definitive IMRT using an isotropic five-millimetre geometrical margin around the macroscopic tumor to create the high-dose CTV (CTV-P1) [35]. It was demonstrated that the transition towards consensus GTV-CTV-P1 margins did not influence local tumour control, most local recurrences being inside the CTV-P1 and covered by the prescription dose. The GTV is usually well defined on imaging, while the patterns of spread of infra-clinical disease cannot be imaged. Therefore, this geometrical approach seemed reasonable for the CTV-P1 but studies on patterns of relapses are still warranted to assess the patterns of relapse after a geometrically expanded CTV-P2. The 2018 international consensus was challenged with respect to the narrative expert-based consensus [5], and lack of reported agreement rates (in contrast to a consensus on nasopharyngeal cancers [14,36], while the GORTEC methodology on the CTV-P2 was obtained using a “formalised consensus” methodology [[9], [10], [11], [12], [13]].

## 5 Conclusion

The GORTEC proposal on the delineation of CTV-P2, obtained by a “formalised consensus” methodology, provides an adaptation of the 2018 international consensus. It suggests to apply a “geo-anatomical” approach to obtain the low risk CTV-P2 by further customising the geometric expansions based on anatomical barriers, dissemination routes and accounting for the proximity of organs at risk.

## Author contributions

All authors contributed to either study conception and rounds and writing + revising manuscript. Or rounds + revising manuscript.

## Funding

None.

## Declaration of Competing Interest

The authors declare that they have no known competing financial interests or personal relationships that could have appeared to influence the work reported in this paper.
