# Supplementary material for: A GORTEC survey on low-risk CTV-P2 delineation in head and neck cancers
Source: Clin Transl Radiat Oncol. 2025 May 20;53:100980. doi: 10.1016/j.ctro.2025.100980 (PMC12152911; doi:10.1016/j.ctro.2025.100980)
Supplement: Supplementary Data 1 [file mmc1.docx]

**Anatomical delineation of low-risk CTV P2** **that was routinely used until 2018: Anatomical structures and compartments corresponding to tumor extension pathways to be delineated (table established based on the 3 cited references).**CTV P2 is defined by first delineating the gross tumor volume (GTV) and then adding the various at-risk structures or spaces while respecting anatomical barriers and excluding air. These structures and spaces may be partially delineated depending on their risk of involvement. A partial delineation extending 1 cm beyond the GTV (2 cm or more if the estimated risk is high, i.e., poorly defined tumor, large tumor, lymphatic emboli, perineural infiltration, etc.) is considered an acceptable compromise.

1. Lapeyre M, Henrot P, Alfonsi M, Bardet E, Bensadoun RJ, Dolivet G, et al. [Propositions for the selection and the delineation of peritumoral microscopic disease volumes in oral cavity and oropharyngeal cancers (lymph nodes excluded)]. Cancer Radiother. juin 2005;9(4):261‑70.

2. Lapeyre M, Bailly C, Toledano I, Montalban A, Russier M. [Hypopharynx and larynx cancers: propositions for the selection and the delineation of peritumoral microscopic disease volumes (lymph nodes excluded)]. Cancer Radiother. nov 2010;14 Suppl 1:S43-51.

3. Eisbruch A, Foote RL, O’Sullivan B, Beitler JJ, Vikram B. Intensity-modulated radiation therapy for head and neck cancer: emphasis on the selection and delineation of the targets. Semin Radiat Oncol. juill 2002;12(3):238‑49.

| **Pathways of tumor extension based on sub-localization allowing the delineation of the low-risk CTV P2** | |
| --- | --- |
| **Subsites** | **Oral Cavity** |
| Floor of the Mouth | - Inferiorly and in depth: Includes the sublingual gland, Wharton’s duct medially, and the genioglossus and geniohyoid muscles. The mylohyoid muscle serves as a barrier, but advanced lesions may infiltrate it. - Anteriorly and laterally: Involvement of the gingiva and periosteum of the mandible. The periosteum is a barrier to mandibular invasion, but once involved, tumors tend to spread along it, with bone invasion occurring at a later stage. The extension may progress along the alveolar ridge before reaching the bone. - Posteriorly: Tumor extension can spread toward the pelvilingual sulcus and along the extrinsic muscles of the tongue (genioglossus, laterally styloglossus, hyoglossus, and palatoglossus muscles), eventually reaching the soft tissues of the neck posterior to the mylohyoid muscle, near the mandibular angle and toward the submandibular gland. - Medial extension (for lateral tumors): Progression toward the pelvilingual sulcus and tongue muscles (hyoglossus and intrinsic tongue muscles). |
| Mobile Tongue | - Early extension occurs superficially before progressively infiltrating the intrinsic tongue muscles (longitudinal, transverse, and vertical fibers) and deeper tongue muscles (genioglossus, laterally styloglossus and hyoglossus). - Anterior and lateral extension occurs through the pelvlingual sulcus into the floor of the mouth. - Lesions in the posterior third spread toward the tongue muscles (hyoglossus and styloglossus), then toward the floor of the mouth, the anterior tonsillar pillar (palatoglossus muscle), and the base of tongue, involving the glossotonsillar sulcus. The extension pattern of posterior mobile tongue lesions resembles that of base of tongue cancers. |
| Buccal Mucosa (Inner Cheek Surface) | - Superficial lesion extension spreads contiguously across the inner cheek surface, with the labial mucosa being rarely affected. - Lateral extension: Involves cheek thickness, with infiltration of the buccinator muscle, subsequently affecting deeper muscle layers and extending to the skin. - Posterior extension: Involves the buccal fat pad (buccinator space), pterygomandibular raphe, mandibular ramus, medial pterygoid muscle, then extends to the parotid space, masseter muscle, and eventually reaches the skull base via the pterygopalatine fossa and foramen rotundum. - Cranio-caudal extension: Spreads toward the superior and inferior gingivobuccal sulci and subsequently involves the gingiva. |
| Lower Gingiva | - Early extension spreads toward the periosteum and subsequently to the mandible. - Medial or posterior extension: Progression into the floor of the mouth. - Lateral extension: Spreads to the gingivobuccal sulcus and adjacent buccal mucosa. - Deep invasion: Tumor may extend into the mandibular medullary cavity via the alveolar ridge, involving the inferior alveolar nerve, which ascends toward the mandibular foramen and may ultimately reach V3 (mandibular nerve). |
| Upper Gingiva | - Medial extension: Progression toward the hard palate and subsequently the soft palate. - Lateral and anterior extension: Spreads toward the superior gingivobuccal sulcus and buccal mucosa.  - Posteriorly: Toward the intermaxillary commissure (via the pterygomaxillary raphe), the buccal space, and then the parotid space. - Superiorly and in depth: May extend toward the bone and invade the maxillary sinus. |
| Hard Palate | - Extension occurs progressively toward the contralateral hard palate. - Posterior extension: Toward the soft palate. - Lateral extension: Toward the superior gingivobuccal sulcus and the buccal mucosa. - Deep infiltration may extend to the bone and invade the maxillary sinus.. |
| Retromolar Trigone | -Laterally: Toward the buccal mucosa (inner cheek surface, extending anteriorly) and posteriorly toward the buccinator muscle and adipose spaces. - Medially: Toward the anterior tonsillar pillar (palatoglossus muscle) and then the soft palate. - Inferiorly: Toward the lower gingiva and the floor of the mouth. Periosteal invasion of the mandible occurs rapidly, but osseous invasion of the mandible is a late manifestation. - Superiorly and anteriorly: Toward the upper gingiva and maxillary bone. A classic pattern of superior extension follows the pterygomandibular raphe. Once breached, the tumor progresses contiguously toward the pterygoid process and the adipose spaces. - Posteriorly: Toward the pterygomaxillary fossa with involvement of the medial pterygoid muscle. Tumor extension then progresses to the posteroinferior portion of the parapharyngeal space and the posterior wall of the maxillary sinus. Subsequently, invasion extends to the pterygopalatine fossa and then to the skull base (foramen rotundum). |

| **Subsites** | **Oropharynx** |
| --- | --- |
| Anterior Tonsillar Pillar | - Anteriorly and laterally: Toward the retromolar trigone (intermaxillary commissure), posterior gingiva, gingivobuccal sulcus, and the posterior portion of the buccal mucosa. Invasion may extend into the deep spaces and the lower part of the infratemporal fossa. - Anteriorly: Toward the posterior floor of the mouth and the mobile tongue (lingual junction zone). - Superiorly: Through the palatoglossus muscle, toward the hard palate and the upper gingiva, with possible infiltration of the pterygoid muscles and sometimes the pterygoid process. - Medially: Toward the lingual junction zone, the glosso-tonsillar sulcus, and the base of the tongue |
| Tonsil | -Anteriorly: Toward the palatoglossus muscle (anterior pillar), then the intermaxillary commissure, posterior floor of the mouth, lingual junction zone, and adjacent mobile tongue. - Medially: Toward the glossotonsillar sulcus and the base of the tongue. - Inferiorly: Into the deep portion of the glossotonsillar sulcus and toward the confluence of the three mucosal folds. Infiltration progresses deeply downward via the parapharyngeal space toward the tongue through the styloglossus muscle. - Posteriorly: Toward the posterior tonsillar pillar (palatopharyngeus muscle), then the lateral and posterior pharyngeal walls. - Superiorly: Toward the nasopharynx (tensor and levator veli palatini muscles) or the soft palate via the tonsillar arch. - Deep superior extension: Toward the pharyngeal aponeurosis, which is rapidly invaded. The tumor then involves the pharyngeal musculature (superior and middle constrictor muscles). Progressively, the tumor breaches the peripharyngeal aponeurosis, invading the styloglossus muscle and the parapharyngeal space, extending superiorly toward the styloid process and then following V3 to the foramen ovale. Advanced tumors may involve the pterygopalatine fossa with extension to the foramen rotundum. |
| Posterior Tonsillar Pillar | - Medially: Toward the posterior pharyngeal wall. - Anteriorly: Toward the tonsil.  - Superiorly: Soft palate (tensor and levator veli palatini muscles) and nasopharynx. - Posteriorly and superiorly: Retropharyngeal space. - Inferiorly: Pharyngoepiglottic fold along the palatopharyngeus muscle up to the junction of the three folds, then the piriform sinus. - Laterally: Parapharyngeal space up to the foramen ovale. |
| Soft Palate | - - Medially: Contralateral soft palate. - Laterally and inferiorly: Tonsil and the superior portion of the tonsillar pillars. - Superiorly: Nasopharynx via the tensor and levator veli palatini muscles - - Laterally, the ipsilateral medial pterygoid muscle and the pterygomaxillary fossa and parapharyngeal space, extending superiorly to the foramen ovale and the trigeminal ganglion. - Anteriorly and laterally: Retromolar trigone. |
| Glossotonsillar Sulcus | - Laterally: Towards the anterior pillar. - Superiorly: Towards the tonsil, then the posterior pillar medially. - Medially: Towards the base of the tongue. - Anteriorly and laterally: Towards the mobile tongue and the floor of the mouth (posterior third). - Inferiorly: Extending to the vallecula and the junction of the three folds. - Deep laterally and posteriorly: Towards the parapharyngeal space. |
| Base of the Tongue | - - Medially: Towards the contralateral base of the tongue. - Inferiorly: Towards the vallecula. - Anteriorly: Towards the mobile tongue and then the floor of the mouth. - Laterally: Towards the glossotonsillar sulcus, then towards the tonsil and the anterior and posterior pillars (palatoglossus and styloglossus muscles). Bone invasion can extend to the styloid muscles in advanced tumors. - Deeply: Towards the extrinsic tongue muscles (genioglossus, palatoglossus, hyoglossus), then towards the mylohyoid muscle. - Posteriorly and deeply: Possible extension beyond the mylohyoid muscle, in contact with the submandibular gland. |
| Vallecula | - - Anteriorly and superiorly: Towards the base of the tongue. - Medially: Extension to the contralateral vallecula. - Laterally and inferiorly: Invasion of the junction of the three folds, then the superior part of the piriform sinus and the lateral pharyngeal wall. - Posteriorly: Towards the lingual surface of the epiglottis and inferiorly towards the pre-epiglottic space. |
| Posterior Oropharyngeal Wall | - Laterally and craniocaudally: Extension along the posterior pharyngeal wall. - Laterally: Towards the posterior tonsillar pillar, then the parapharyngeal space. - Superiorly: Retropharyngeal space extending to the nasopharynx. - Inferiorly: Towards the junction of the three folds and the anterior angle of the piriform sinus. - Towards the prevertebral muscles: Very rare extension due to the difficult to penetrate anterior vertebral ligament acting as a barrier |

| **Subsites** | **Larynx** |
| --- | --- |
| Suprahyoid Epiglottis | - - Anteriorly: The lingual surface of the epiglottis, the vallecula, and then the base of the tongue, infiltrating deeply into the superior portion of the pre-epiglottic space by crossing inferiorly through the hyoepiglottic ligament. - Laterally: Towards the region of the three folds, the aryepiglottic fold, the anterior angle of the piriform sinus, and then the lateral pharyngeal wall. ; - - Inferiorly along the epiglottis and the pre-epiglottic space |
| Subhyoid Epiglottis | - - Anteriorly through the epiglottis into the pre-epiglottic space via the epiglottic pores or by lateral infiltration of the epiglottis. - Inferiorly towards the anterior commissure and the thyroepiglottic ligament. The extension then progressively involves the glottis and subglottis. Lesions can also extend anteriorly towards the pre-epiglottic space and progress superiorly, transfixing the hyoepiglottic ligament and invading the vallecula and the base of the tongue. - Laterally towards the vestibular fold, ventricles, and aryepiglottic folds. - Superiorly towards the free edge of the epiglottis |
| Vestibular fold | - - Superiorly towards the aryepiglottic fold and the arytenoid. - Anteriorly towards the base of the epiglottis. - Inferiorly towards the ventricles. - Posteriorly towards the posterior commissure. - Laterally towards the paraglottic space, intrinsic laryngeal muscles, pharyngolaryngeal wall, and thyroid cartilage. |
| Ventricle | - - Laterally towards the paraglottic space, intrinsic laryngeal muscles, pharyngolaryngeal wall, and thyroid cartilage. - Anteriorly towards the edge of the epiglottis and the pre-epiglottic space (superiorly). - Inferiorly towards the vocal cords and subglottis after rupture of the elastic cone. - - Superiorly towards the vestibular fold then aryepiglottic fold and the arytenoid. |
| Arytenoid or aryepiglottic fold | - Inferiorly towards the cricoarytenoid muscle, leading to hemilarynx fixation. Reduced hemilaryngeal mobility may also be associated with involvement of the superior laryngeal nerve passing through the thyrohyoid membrane or by invasion of the paraglottic space. - Extension to the cartilages (thyroid, epiglottis, cricoid) occurs at a later stage. - Laterally towards the piriform sinus. - Superiorly and anteriorly towards the epiglottis, then the base of the tongue and the valleculae, where the three folds converge . |
| Vocal cord | - - Anteriorly and superficially towards the anterior commissure, then extending to the contralateral vocal cord along Broyle’s ligament. - Laterally towards the floor and the depth of the ventricle, the paraglottic space, intrinsic laryngeal muscles, pharyngolaryngeal wall, and thyroid cartilage. - Posteriorly towards the cricoarytenoid joint and the posterior commissure. - Inferiorly towards the lower surface of the vocal cord and subglottis along the elastic cone. - Superiorly towards the supraglottic region via the ventricle and vestibular fold. . |
| Anterior commissure | - Towards Broyle’s ligament, infiltrating the pre-epiglottic space by crossing the thyroepiglottic ligament superiorly or extending anteriorly towards the thyroid cartilage, then into the prelaryngeal tissues by crossing the cricothyroid ligament inferiorly. - Inferiorly towards the subglottis. - Posteriorly towards the vocal cords. - Anteriorly, crossing the thyroid cartilage and extending into the neck tissues and thyroid gland. |
| Subglottis | - Primarily towards the cricoid ring. - Laterally and anteriorly through the cricothyroid membrane into the neck and towards the thyroid gland, and laterally and posteriorly towards the esophageal inlet. - Inferiorly towards the trachea. - Superiorly towards the glottic plane. |

| **Subsites** | **Hypopharynx** |
| --- | --- |
| Piriform Sinus: Medial Wall and Anterior Angle | - Anteriorly and medially to infiltrate the arytenoid, then the other laryngeal cartilages, the paraglottic space, and the pre-epiglottic region. - Superiorly, towards the junction of the three pharyngeal folds and along the posterior pillar of the tonsil, extending to the soft palate and then to the skull base via the longitudinal pharyngeal muscles (palatopharyngeus, stylopharyngeus, and salpingopharyngeus). - Inferiorly, along the cricothyroid membrane and extending towards the esophageal inlet. - Laterally, extending towards the lateral wall of the piriform sinus. - Posteriorly, towards the retrocricoid region, possibly reaching the contralateral piriform sinus. |
| Piriform Sinus: Lateral Wall | - Laterally, extending to the posterior portion of the thyroid cartilage, the lateral pharyngeal wall, and the soft tissues of the neck. - Anteriorly, involving the paraglottic space. - Medially, extending towards the medial wall of the piriform sinus and the lateral aspect of the larynx. - Superiorly, along the posterior pillar of the tonsil, extending to the soft palate and then towards to the skull base via the longitudinal pharyngeal muscles (palatopharyngeus, stylopharyngeus, and salpingopharyngeus). - Inferiorly, extending to the esophageal inlet. |
| Lateral Pharyngeal Wall of the Hypopharynx | - Laterally, extending towards the pharyngeal constrictor muscles, the cervical soft tissues, the vascular space, and the thyroid gland. - Posteromedially, extending towards the posterior pharyngeal wall. - Anteriorly, extending towards the thyroid lamina. - Superiorly, extending towards the pharyngoepiglottic fold, then secondarily to the vallecula and medially and anteriorly to the piriform sinus. The superior extension continues along the longitudinal pharyngeal muscles to their insertions on the styloid process, soft palate, and Eustachian tube. It may also spread along the oropharyngeal pharyngeal wall, reaching the nasopharynx. - Inferiorly, extending to the esophageal inlet. |
| Posterior Hypopharyngeal Wall | - Superiorly, extending along the oropharyngeal pharyngeal wall, possibly reaching the nasopharynx. The superior extension may also progress along the longitudinal pharyngeal muscles to their insertions on the styloid process, soft palate, and Eustachian tube. - Superiorly and laterally, extending along the posterior pillar of the tonsil, reaching the soft palate via the palatopharyngeus muscle. - Posteriorly, extending towards the prevertebral muscles. - Anterolaterally, extending towards the lateral pharyngeal wall. - Medially, crossing the midline, leading to circumferential involvement. - Inferiorly, extending to the esophageal inlet. |
| Retrocricoid Region | - Inferiorly, extending towards the esophageal inlet. - Anteriorly, infiltrating the cricoid cartilage and potentially extending into the endolarynx via the inter-arytenoid region. - Superiorly, extending towards the arytenoids and the aryepiglottic folds. - Laterally, spreading towards the piriform sinuses and the medial wall of the piriform sinus. |
