# Supplementary material for: A GORTEC survey on low-risk CTV-P2 delineation in head and neck cancers
Source: Clin Transl Radiat Oncol. 2025 May 20;53:100980. doi: 10.1016/j.ctro.2025.100980 (PMC12152911; doi:10.1016/j.ctro.2025.100980)
Supplement: Supplementary Data 2 [file mmc2.docx]

Figure : Example of CTV P2 delineation for a T2 tonsil tumor using the anatomical method that was routinely used until 2018. The GTV is shown in red, and the CTV P2 in yellow. Distances are indicated in white on a blue background (delineation established based on the 3 cited references).

| 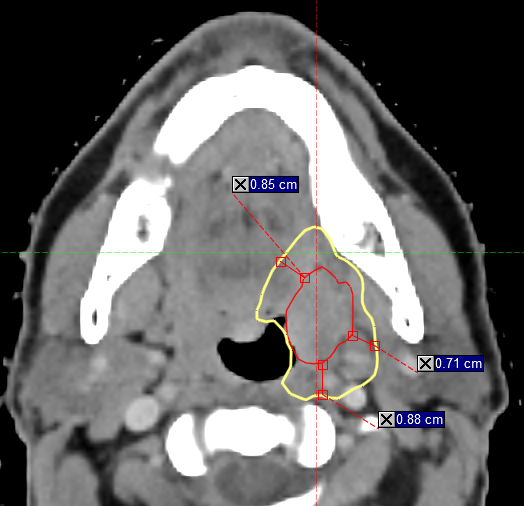 | |
| --- | --- |
| 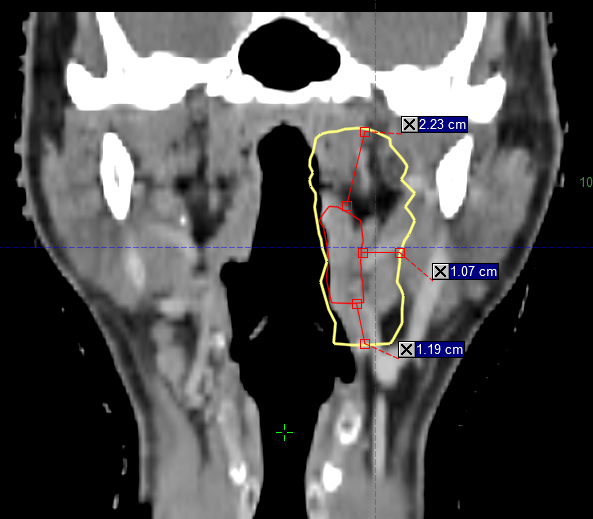 | 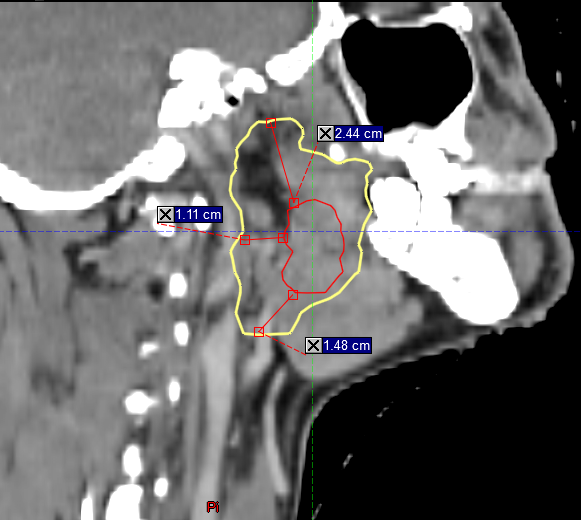 |

1. Lapeyre M, Henrot P, Alfonsi M, Bardet E, Bensadoun RJ, Dolivet G, et al. [Propositions for the selection and the delineation of peritumoral microscopic disease volumes in oral cavity and oropharyngeal cancers (lymph nodes excluded)]. Cancer Radiother. juin 2005;9(4):261‑70.

2. Lapeyre M, Bailly C, Toledano I, Montalban A, Russier M. [Hypopharynx and larynx cancers: propositions for the selection and the delineation of peritumoral microscopic disease volumes (lymph nodes excluded)]. Cancer Radiother. nov 2010;14 Suppl 1:S43-51.

3. Eisbruch A, Foote RL, O’Sullivan B, Beitler JJ, Vikram B. Intensity-modulated radiation therapy for head and neck cancer: emphasis on the selection and delineation of the targets. Semin Radiat Oncol. juill 2002;12(3):238‑49.
